# Early Nutrition Must Be Safe and Should Have Positive Impacts on Long-Term Health

**DOI:** 10.3390/nu15122645

**Published:** 2023-06-06

**Authors:** Nadja Haiden, Ferdinand Haschke

**Affiliations:** 1 Department of Neonatology, Kepler University Hospital, 4020 Linz, Austria; 2 Department Pediatrics, Paracelsus Medical University, 5020 Salzburg, Austria; fhaschk@gmail.com

The Special Issue entitled ‘The Role of Feeding Practice and Early Nutrition in Infant Growth, Metabolism and Body Composition’ examines the long-term outcomes of early nutrition in both preterm and term infants. The issue covers important topics related to the nutrition of preterm infants, such as the safety of docosahexaenoic acid (DHA) supplementation, the optimal timing for introducing solid foods, the follow-up of vitamin D and iron nutritional status, early probiotic supplements and their effects on morbidity, as well as cognitive and neuropsychological development after post-discharge fortification of a mother’s milk. Furthermore, the issue compares the growth and iron nutrition status of young children born at term who received cow’s milk, non-fortified formulas, or fortified young child formula.

The omega-3 long-chain polyunsaturated fatty acid DHA (22:6 n-3) is critical for neurodevelopment in infants. It is the major fatty acid in the brain and is transferred from the mother to the fetus during pregnancy, primarily during the last trimester. However, infants born prematurely miss out on this important transfer of DHA, which can affect their neurodevelopment. The topic of DHA supplementation for preterm infants has been reviewed by ESPGHAN [1] who concluded that supplementation has modest and transient effects on neurodevelopmental outcomes, but may help achieve intakes similar to intrauterine accretion rates. Late in 2022, a study showed [2] that preterm infants who received DHA supplementation until 36 weeks of postmenstrual age had slightly higher full-scale intelligence quotient scores at 5 years of age than those in the control group. A DHA intake of 30 to 65 mg/kg/d (approximately 0.5% to 1% of total fatty acids) is now recommended by ESPGHAN, assuming sufficient intake of arachidonic acid (ARA) is provided. Formulas for preterm infants and human milk fortifiers are now supplemented with DHA and ARA.

However, the safety of providing DHA supplements to preterm infants has been questioned in some studies. One systematic review of children born at term [3] showed inconsistent effects of n-3 fatty acids on obesity and blood pressure. Another study on preterm infants showed [4] that girls who received DHA-supplemented formula were heavier and had greater skin fold thicknesses at 10 years of age. Preterm infants may also be at higher risk of cardiometabolic disease in later life. [5]. One study [6] showed that DHA supplementation in infancy was associated with higher systolic blood pressure at 10 years of age, but only in girls. However, a multicenter randomized controlled trial (DINO trial) on the supplementation of infants born <33 weeks’ gestation (*n* = 657) with higher dose DHA showed no effect on weight, height, body composition, and blood pressure at 7 years corrected gestational age. Interestingly, the high-DHA group infants were more likely to be classified as obese, but this finding needs to be interpreted with caution because it was a secondary outcome variable and the number of children classified as obese was small [7].

Probiotics are used to reduce mortality and morbidity such as NEC and sepsis in very low-birth-weight infants during their early life [8]. Although there is no clear evidence on which strain is the most effective, dosage, and the start and end of probiotic administration, and the ideal number of living germs administered, there is a consensus that probiotics are effective in preventing diseases and reducing mortality [9]. However, in the present study [10], the authors investigated if the administration of a product with three combined strains of one billion CFUs of Bifidobacterium infantis Bb-02 (DSM 33361), Bifidobacterium lactis (BB-12), and Streptococcus thermophilus (TH-4) is effective to improve feeding tolerance in infants between 28 and 32 weeks of gestation and compared the cohort with a historic control group of a period before probiotics were implemented as the standard of care. The risk of the primary composite outcome of death, sepsis, or NEC, adjusted for gestational age and birth weight (Fenton z-score), seemed lower in the probiotics group (4.3% vs. 9.2%, *p* = 0.08; aRR 0.44, 95% CI 0.18 to 1.08), which is in line with the current literature. In addition, probiotics administration improved feeding tolerance, as probiotics were associated with a shorter time to full enteral feeding, more favorable growth, less need for an abdominal X-ray, and shorter duration for antibiotics treatment. Future trials should investigate if these effects are reproducible in the group of more immature infants with a gestational age between 22 and 28 weeks.

In 2022 Haiden et al. [11] showed in a prospective randomized two-group trial that the timepoint of early (10th–12th week of life corrected for term) or late (16th–18th week of life corrected for term) introduction of solids did not affect the growth of preterm infants in the first year of life. In this first high-quality trial in a high-income country setting, infants with a mean gestational age of 27 + 1 weeks at birth had to adhere to a “ready to use” complementary feeding protocol, enabling standardized conditions for the calculation of nutrient intake. Secondary outcomes of the study were published in this Special Issue and provided data on vitamin D [12] and iron status [13] during the first year of life. Infants were supplemented with 625 IE/d in addition to the intake of food. At the end of the first year of life, 89% in the early and 81% in the late group developed Vit D deficiency as defined by a Serum 25-OH Vitamin D below 50 nmol/L. Although the daily administration of Vitamin D supplements was self-reported by the parents, these results confirm that fast-growing former preterm infants might have higher Vit D needs than term infants.

Data on iron status after discharge suggested that at the age of 6 weeks after term, 56% of the infants in the early and 63% of the late group were iron deficient, indicating that infants, for the first months after discharge, are susceptible to iron deficiency, although they were supplemented with approx. 4 mg/kg/d [14,15]. This can be explained by various circumstances still lingering from their early neonatal period and their stays in the intensive care unit such as high volumes of phlebotomy loss, physiologic 3-month anemia, protein deficiency, and higher needs due to catch-up growth. Iron deficiency improved during the first year of life under supplementation, and at 6 and 12 months corrected for term less than 10% of the infants were iron deficient. These results indicate that especially early sufficient iron supplementation is very important in preterm infants as iron deficiency can cause poor growth, abnormal neurological reflexes at term, and impaired neurodevelopmental outcome [16,17]. However the timepoint of the introduction of solids itself neither affected Vit-D status nor iron status but this was a secondary outcome analysis and adequate powered studies have to confirm these results.

Small preterm infants (gestational age < 32 weeks) are at increased risk of poor neurodevelopmental outcomes in childhood [18]. Higher protein and energy intakes, as well as higher growth rates, have been positively correlated with higher cognitive and motor scores [19]. Most nutrition intervention studies were performed during the early postnatal period in the neonatal intensive care unit (NICU) where a mother‘s milk and/or donor human milk (DHM) mixed with human milk fortifiers or preterm formulas were provided. It is well established that the use of human milk during the early feeding period provides several short- and long-term benefits when compared with formula feeding. However, controlled studies on post-discharge nutrition in preterm infants and long-term IQ development were not found in the literature. The follow-up study by Klamer [20] now closes that knowledge gap: it included follow-up on cognitive and neuropsychological development until 6 years corrected age in 214 small preterm infants, including 141 breastfed infants randomized to mother’s milk with or without fortification and 73 infants fed a preterm formula (from discharge to 4 months corrected age). Post-discharge fortification of a mother’s milk did not improve either full-scale intelligence quotient with a median of 104 vs. 105.5 (*p* = 0.29) and subdomain scores. Compared to the formula group, the mother’s milk group had a significantly better verbal comprehension score with a median of 110 vs. 106 (*p* = 0.03) and a significantly better motor skills score (*p* = 0.01). This study is helpful to design further fortification studies in small preterm infants.

The World Health Organization recommends continuing breastfeeding beyond one year of age, yet in both developed and developing countries, most toddlers are introduced to cow’s milk or milk-based formulas between the ages of 1 and 3 years. In recent years, young child formulas (also known as growing-up milks for children aged >1 year) [21] have become popular in many countries due to their lower protein content compared with cow’s milk. High concentrations of insulinogenic amino acids in early life may contribute to later obesity, hence the lower protein content in these formulas. Additionally, young child formulas are fortified with micronutrients to ensure optimal growth and development. A recent randomized controlled trial conducted in Australia and New Zealand [22] compared the body composition of toddlers who consumed a lower protein young child formula (1.7 g/100 mL) or cow’s milk (3.1 g/100 mL) during the second year of life. Toddlers receiving the lower young child formula had a lower percentage of body fat (*p* < 0.04) and lower fat mass (*p* < 0.01) at 2 years of age. However, the results concerning the effects of young child formulas on other health outcomes in toddlers have been inconclusive, in particular in developing societies. A contemporary synthesis of studies investigating the effects of consuming fortified milk beverages (compared to cow’s milk or unfortified comparator formula) on growth and/or nutritional status in children 1–3 years of age [23] indicates higher weight in lower-income countries, in particular in studies with intervention periods >6 months. An important finding of this synthesis is that young children consuming iron-fortified milk have better iron nutritional status, with higher concentrations of hemoglobin (MD = 3.76 g/L (95% CI 0.17, 7.34), *p* = 0.04) and ferritin (MD = 0.01 nmol/L (5% CI 0.00, 0.02]) *p* = 0.02). Although young child formulas can improve the nutrition of toddlers, it is important for the industry to adhere to global and local recommendations that discourage the advertisement of these formulas to consumers. 
